# 2D MXenes as Co-catalysts in Photocatalysis: Synthetic Methods

**DOI:** 10.1007/s40820-019-0309-6

**Published:** 2019-09-21

**Authors:** Yuliang Sun, Xing Meng, Yohan Dall’Agnese, Chunxiang Dall’Agnese, Shengnan Duan, Yu Gao, Gang Chen, Xiao-Feng Wang

**Affiliations:** 1 Key Laboratory of Physics and Technology for Advanced Batteries (Ministry of Education), College of Physics, Jilin University, Changchun, 130012 People’s Republic of China; 2 Jilin Key Engineering Laboratory of New Energy Materials and Technologies, Jilin University, Changchun, 130012 People’s Republic of China; 3 A. J. Drexel Nanomaterials Institute and Department of Materials Science and Engineering, Drexel University, Philadelphia, PA 19104 USA; 4 Institute for Materials Discovery, Faculty of Maths and Physical Sciences, University College London, London, WC1E 7JE UK

**Keywords:** MXenes, Photocatalysis, Co-catalyst, Synthetic methods

## Abstract

Since their seminal discovery in 2011, two-dimensional (2D) transition metal carbides/nitrides known as MXenes, that constitute a large family of 2D materials, have been targeted toward various applications due to their outstanding electronic properties. MXenes functioning as co-catalyst in combination with certain photocatalysts have been applied in photocatalytic systems to enhance photogenerated charge separation, suppress rapid charge recombination, and convert solar energy into chemical energy or use it in the degradation of organic compounds. The photocatalytic performance greatly depends on the composition and morphology of the photocatalyst, which, in turn, are determined by the method of preparation used. Here, we review the four different synthesis methods (mechanical mixing, self-assembly, in situ decoration, and oxidation) reported for MXenes in view of their application as co-catalyst in photocatalysis. In addition, the working mechanism for MXenes application in photocatalysis is discussed and an outlook for future research is also provided.

## Highlights

- Two-dimensional transition metal carbides/nitrides (MXenes) as co-catalysts were summarized and classified according to the different synthesis methods used: mechanical mixing, self-assembly, in situ decoration, and oxidation.
- The working mechanism for MXenes application in photocatalysis was discussed. The improved photocatalytic performance was attributed to enhancement of charge separation and suppression of charge recombination.

## Graphical abstract

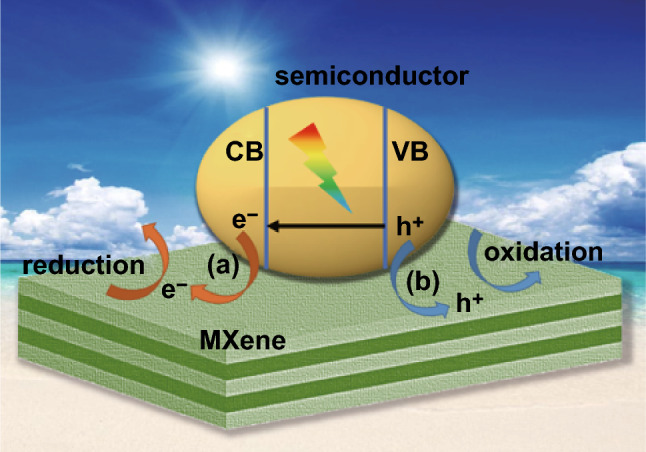

## Introduction

Energy shortage and environmental pollution have become the two major issues faced by humanity due to limited fossil fuel resources and increasing consumption. Developing sustainable and clean energy is the key to addressing these two problems [1–15]. In being clean and inexhaustible, solar energy shows great potential to be one of the most promising future energy sources. Solar energy can be exploited in photovoltaic technologies [16], CO_2_ photoreduction [17, 18], N_2_ photo-fixation [19], degradation of organic compounds [20–26], and photocatalytic water splitting [27]. In renewable hydrogen fuel-based photocatalytic water-splitting systems [28–30], photocatalysts play a critical role [31, 32]. Photo-catalyzed solar energy conversion can be divided into three steps: (1) light absorption, (2) charge separation and transfer, and (3) surface reaction. Any improvement on each of these steps will contribute to enhancing the total conversion efficiency. Conventional photocatalysts such as TiO_2_, g-C_3_N_4_, and CdS demonstrate low photocatalytic efficiency due to rapid charge recombination in these materials. Using noble metals such as Pt, Ru, and Pd as co-catalysts will increase cost, although such materials can enhance charge separation ability and suppress recombination of charges. A co-catalyst that is both efficient and cheap is thus urgently needed to promote the development of photocatalysis.

**Fig. 1:**
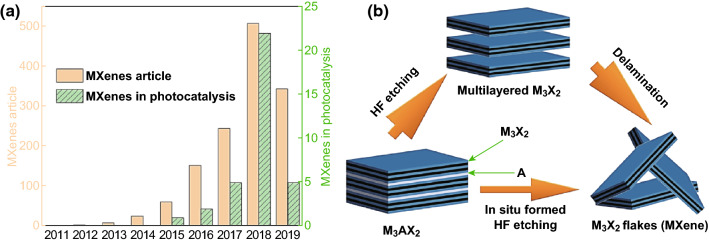
**a** The rapid expansion of 2D MXenes materials and **b** the most widely used methods to synthesize MXenes

MXenes, comprising transition metal carbides, nitrides, and carbonitrides, are a new family of two-dimensional (2D) materials that have attracted much attention in recent years [2]. The general formula of MXene is M_*n*+1_X_*n*_ (*n* = 1, 2, 3), where M represents a transition metal, such as Sc, Ti, Zr, Hf, V, Nb, Ta, and Mo, while X represents C and/or N. Owing to their unique structure and superior photoelectronic properties, layered structure MXenes show various potential applications in different areas, such as energy storage [3, 33–38], electromagnetic interference shielding [39, 40], gas sensors [41], wireless communication [42], water treatment [43, 44], solar cells [45–47], and catalysis [41, 48–51]. 2D MXenes are being increasingly studied in the past few years, as evidenced by the rapidly increasing number of scientific articles published per year (Fig. 1a). MXenes are usually synthesized by selectively etching the A layer from MAX phases, which constitute a family of tertiary ductile ceramics, where the A layer is made of an element such as Al, Ga [52], or Si [53]. After selective etching of the A layer, 2D MX layers with surface functional groups (–O, –OH, –F, or a mixture of several groups denoted as T_*x*_) are left. The most widely used methods for selective etching are wet chemical HF etching and in situ HF etching (using a mixture of acids and fluoride salts), although other routes using tetramethylammonium hydroxide (TMAOH) [54, 55], electrochemical [56, 57], or etching with NaOH [58], and ZnCl_2_ [49]) have also been explored. Generally, multilayered MXenes are produced by HF etching, whereas single or few-layered MXene flakes are obtained by in situ HF etching or through delamination of a multilayered MXene by intercalation of large organic molecules (Fig. 1b). The etching methods of Ti_3_C_2_T_*x*_ MXene, which is the first discovered and the most studied MXene, have been reviewed elsewhere [59, 60].

In view of the rapid development in the application of 2D MXenes, several reviews on their synthesis [59–61], and application in energy storage [33, 48, 62] and catalysis [51] have been reported. MXenes are promising for application in photocatalysis [63] because of their large surface area, good conductivity, presence of a sufficient number of active sites, and containing suitable elements for effective photocatalysis, but they cannot be directly used as photocatalysts since MXenes are generally not semiconductors [51, 62]. Although there are some MXene semiconductors that have been predicted theoretically [64–68], these have not yet been experimentally synthesized. In this review, we give a detailed discussion on MXene as a co-catalyst in photocatalysis and describe the different methods used for the synthesis of MXene-derived photocatalysts, along with problems encountered in this system and a prospective outlook on future research in this field.

## Synthetic Methods for MXenes as Co-catalysts in Photocatalysis

**Fig. 2:**
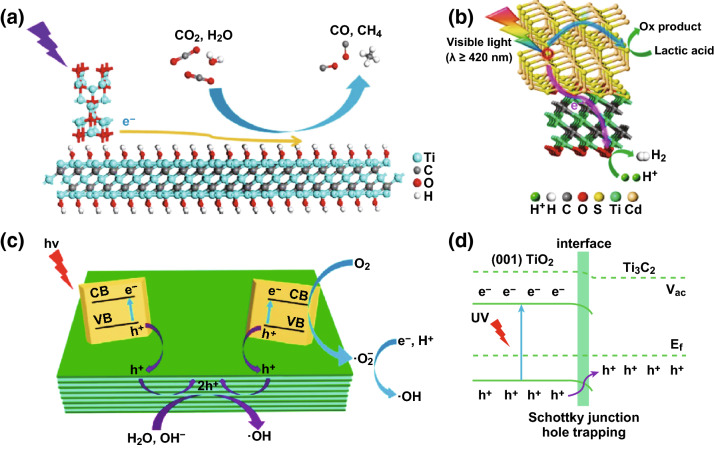
Schematic showing charge separation between MXene co-catalyst and a photocatalyst taken from **a** Ye et al. Reprinted with permission from Ref. [69]. Copyright 2018 John Wiley & Sons. **b** Ran et al. Reprinted with permission from Ref. [70]. Copyright 2017 Nature Publishing Group. **c**, **d** Peng et al. Reprinted with permission from Ref. [71]. Copyright 2016 American Chemical Society

In view of their good conductivity and large surface area, MXenes have been applied in photocatalysis both to replace noble metal co-catalysts and to enhance the charge separation ability of the photocatalyst (Fig. 2). The most common methods used for the preparation of photocatalyst composites include mechanical mixing, self-assembly, in situ decoration and oxidation, or a combination of the three methods.

### Mechanical Mixing and Self-assembly

Mechanical mixing is the easiest method to form photocatalyst composites. Stirring the two components in the liquid phase or grinding of powders can be used for sample preparation. Interestingly, due to electrostatic attraction, photocatalysts with positive charge are easily combined with MXenes whose surfaces are enriched with negative charges, leading to self-assembled photocatalyst composites. In addition, the self-assembling property could be further improved by using other induced techniques simultaneously, where the photocatalysts and co-catalysts are prepared in advance [44].

**Fig. 3:**
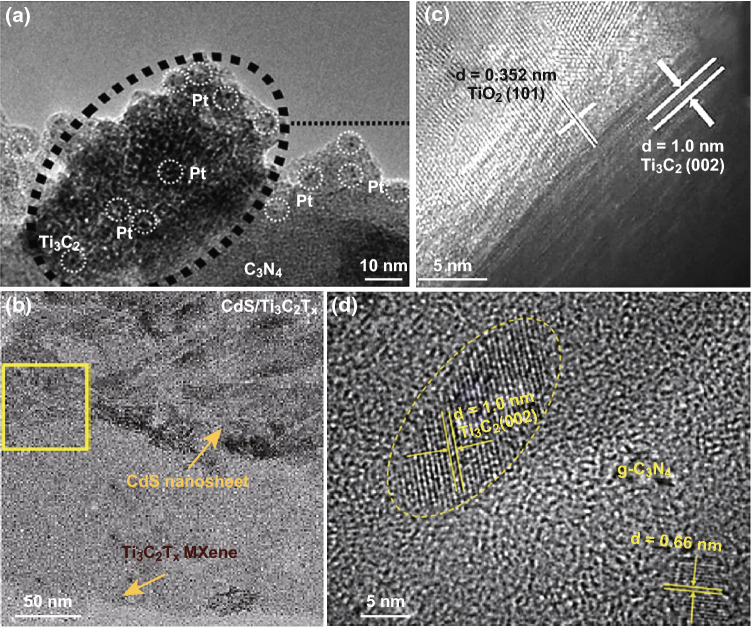
TEM images of photocatalysts combined with a MXene by mechanical mixing taken from **a** An et al. Reprinted with permission from Ref. [72]. Copyright 2018 The Royal Society of Chemistry. **b** Xie et al. Reprinted with permission from Ref. [73]. Copyright 2018 Elsevier. **c** Ye et al. Reprinted with permission from Ref. [69]. Copyright 2018 John Wiley & Sons. **d** Liu et al. Reprinted with permission from Ref. [44]. Copyright 2018 Elsevier

**Fig. 4:**
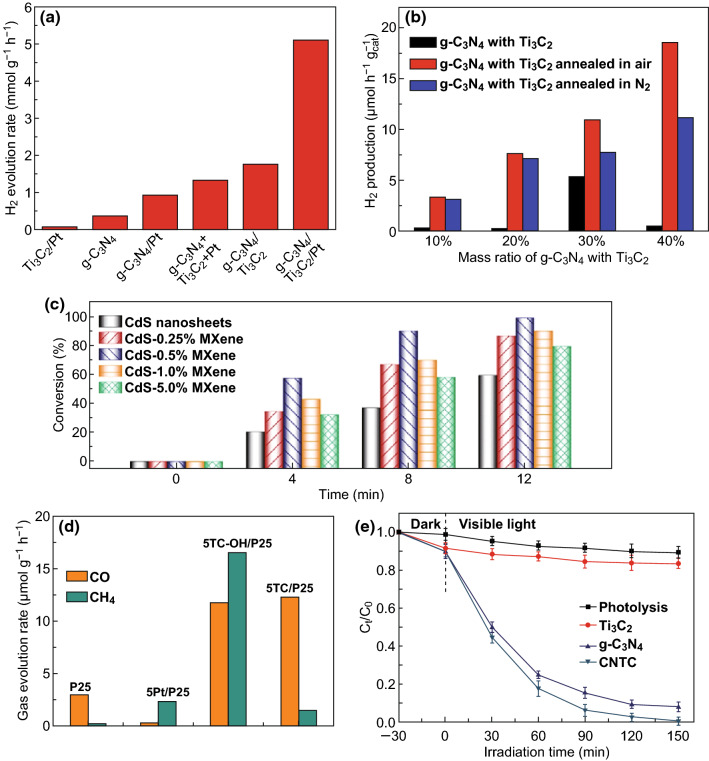
Hydrogen production of different samples taken from **a** An et al. Reprinted with permission from Ref. [72]. Copyright 2018 The Royal Society of Chemistry. **b** Sun et al. Reprinted with permission from Ref. [74]. Copyright 2018 The Royal Society of Chemistry. **c** Photo-degradation of 4-nitroaniline (4-NA) over different samples from Xie et al. Reprinted with permission from Ref. [73]. Copyright 2018 Elsevier. **d** Rates of evolution of CO and CH_4_ over different samples from Ye et al. Reprinted with permission from Ref. [69]. Copyright 2018 John Wiley & Sons. **e** Ciprofloxacin degradation from Liu et al. Reprinted with permission from Ref. [44]. Copyright 2018 Elsevier

An et al. [72] demonstrated that synergetic effects of Ti_3_C_2_ MXene and Pt when used as dual co-catalysts enhanced the photoactivity of g-C_3_N_4_ for hydrogen evolution (Fig. 3a), where HF-etched exfoliated Ti_3_C_2_ and g-C_3_N_4_ were mixed in liquid by stirring followed by photodeposition of Pt on the composites. The photoactivity of the dual co-catalysts-modified photocatalysts (g-C_3_N_4_/Ti_3_C_2_/Pt) was much better than that of Pt- or Ti_3_C_2_-only systems, reaching 5.1 mmol h^−1^ g^−1^ in hydrogen production (Fig. 4a). This enhanced performance was due to the presence of Ti_3_C_2_ MXene that facilitated interfacial charge separation and carrier transport from the conduction band (CB) of g-C_3_N_4_ to Pt. Our group prepared g-C_3_N_4_/Ti_3_C_2_T_*x*_ composites by grinding g-C_3_N_4_ and Ti_3_C_2_T_*x*_ powders together followed by annealing in different gas atmospheres, to tune the surface termination groups (Fig. 4b) [74]. X-ray photoelectron spectroscopy data showed an increase in –O termination groups accompanied by a decrease in –F termination groups on the surface of Ti_3_C_2_. Ti_3_C_2_ with –O termination groups had better photoactivity, revealing that the presence of such groups in Ti_3_C_2_ had a positive effect on hydrogen production by increasing the number of active sites. Moreover, this finding was consistent with density functional theory (DFT) simulation results. The |Δ*G*_H_| of Ti_3_C_2_ with –O terminations was found to be as low as 0.01 eV, which is lower than that of Pt (111). In a similar study, Ye et al. [69] treated HF-etched Ti_3_C_2_ with KOH to convert –F groups into –OH groups, and then combined the KOH-treated Ti_3_C_2_ with TiO_2_ (P25) powder by stirring in water (Fig. 3c). DFT calculations demonstrated that –OH groups played the role of active sites for the adsorption and activation of CO_2_ reduction [69]. Experimentally, the photoactivities for CO_2_ reduction were increased 3 times and 277 times after KOH treatment, for CO and CH_4_, respectively (Fig. 4d). Interestingly, increasing the number of –OH groups not only improved the photo-conversion efficiency but also changed the nature of the products. The –OH groups resulting from KOH treatment provided more active sites for CO_2_ adsorption and enabled greater electron transfer to CO_2_ and facilitated its reduction to CH_4_. Though the surface termination groups can be changed through annealing and KOH treatments, –F groups could not be completely exchanged. More studies to precisely tailor the termination groups need to be carried out in the future.

Xie et al. [73] used an electrostatic self-assembly process to combine positively charged CdS nanosheets and Ti_3_C_2_ nanosheets (possessing negative charge) (Fig. 3b) for CO_2_ reduction (Fig. 4c). Cai et al. [75] synthesized Ag_3_PO_4_/Ti_3_C_2_ by electrostatically driven self-assembly method, which had the advantage of being a mild method that prevented Ti_3_C_2_ from oxidation. The composites showed better performance than reduced graphene oxide (rGO), and this preparation procedure provided a new direction to the preparation of semiconductor-MXene composites. Liu et al. [44] fabricated a 2D layered and stacked g-C_3_N_4_/Ti_3_C_2_ composite by evaporation-induced self-assembly and used it to degrade organic pollutants (ciprofloxacin) (Fig. 3d). Both photogenerated holes and superoxide radicals (·O_2_^−^) resulting from photogenerated electrons played important roles in ciprofloxacin decomposition (Fig. 4f); in this process, self-assembly was an efficient method that allowed intimate mixing of the components in the composite. The sample was also more homogeneous than mechanically mixed ones because of the electrostatic attraction between the charged entities. However, opposite charges on each surface were required for self-assembly, which limited wider application of this process. Therefore, other techniques to induce self-assembly such as evaporation-induced self-assembly were developed to widen the range of application of products [44].

The above-mentioned MXene-based composites prepared by mechanical mixing and self-assembly methods for photocatalysis application are summarized in Table 1. Results from all these works prove that 2D MXene is an efficient additive material to enhance charge separation and charge transfer during photocatalysis. In these two methods, the properties of MXenes are retained by avoiding high temperature and use of other solvents or surfactant. No change in oxidation or surface termination groups occurs in these synthesis methods. Therefore, these two are the easiest and allow synthesis under the mildest conditions.

**Table 1:** MXene-based composites prepared by different synthetic methods for photocatalysis applications

Sample	MXene (synthetic method)	Sample synthesis	Reactant	Sacrificial agent	Rate	Precursor	Refs.
g-C_3_N_4_/3%Ti_3_C_2_/2%Pt	Ti_3_C_2_ flakes (HF 48%, 20 h, 60 °C and H_2_O delamination, 12 h, ultrasonication)	(1) Ti_3_C_2_ stirring dispersions (2) Pt UV deposition	H_2_O	10 vol% triethanolamine (TEOA)	5100 μmol/h/g_cat._	–	An et al. [72]
g-C_3_N_4_/Ti_3_C_2_T_*x*_ (1: 0.3)	Multilayer Ti_3_C_2_ (HF 49%, 24 h)	Grinding in a mortar	H_2_O	10 vol% TEOA	88 μmol/h/g_cat._	–	Sun et al. [74]
CdS/0.5%Ti_3_C_2_T_*x*_	Ti_3_C_2_ flakes (LiF 1 g/HCl 9 M, 24 h, 35 °C)	(1) Ultrasonication (2) Stirring in water	4-NA	40 mg ammonium formate in 30 mL solution	180 mg/L/h	–	Xie et al. [73]
P25/5%Ti_3_C_2_-OH	Multilayer Ti_3_C_2_ (HF 49%, 24 h and KOH 2 M, 4 h)	Stirring in water	CO_2_	–	28.35 μmol/h/g_cat._	–	Ye et al. [69]
a-Fe_2_O_3_/Ti_3_C_2_ (1: 2)	Multilayer Ti_3_C_2_	(1) Stirring in ethanol (2) Ultrasonication	Rhodamine B (RhB)	–	5 mg/L/h	–	Zhang et al. [76]
g-C_3_N_4_/Ti_3_C_2_ (100: 3)	Ti_3_C_2_ flakes (HF 40%, 24 h and H_2_O intercalation, 5 h, ultrasonication)	(1) Ultrasonication (2) Stirring in water at 60 °C	Ciprofloxacin	–	18 mg/L/h	–	Liu et al. [44]
TiO_2_/5%Ti_3_C_2_	Ti_3_C_2_ flakes (LiF 1 g/HCl 6 M, 24 h, 35 °C)	Sonication	H_2_O	25% Methanol	2650 μmol/h/g_cat._	–	Su et al. [77]
			H_2_O			–	
Ag_3_PO_4_/2%Ti_3_C_2_	Ti_3_C_2_ flakes (NaF 3.35 g/HCl 36–38 wt%, 12 h, 60 °C)	(1) Stirring in water with AgNO_3_ (2) Adding Na_2_HPO_4_	Tetracycline hydrochloride (TC-H) etc.	–	192 mg/L/h	–	Cai et al. [75]
3%Ti_3_C_2_/g-C_3_N_4_	Ti_3_C_2_ flakes (LiF 1.5 g/HCl 6 M, 24 h, 35 °C)	(1) Sonication in HCl (2) Stirring	H_2_O	10 vol% TEOA	73.3 μmol/h/g_cat_	–	Su et al. [78]
TiO_2_/0.5%Ti_3_C_2_/1%CoS_*x*_	Multilayer Ti_3_C_2_ (HF 49%, 4 h)	(1) Stirring in 2-methylimidazole (2) Hydrothermal 140 °C for 12 h with thioacetamide	H_2_O	20 vol% methanol	950 μmol/h/g_cat._	Co(NO_3_)_2_, 2-methylimidazole and thioacetamide	Zhao et al. [79]
CdS/MoS_2_/2%Ti_3_C_2_T_*x*_	Ti_3_C_2_ flakes (HF 49%, 72 h, ultrasonication in H_2_O, 2 h)	(1) MoS_2_ synthesis (2) Stirring with Ti_3_C_2_ (3) Add CH_4_N_2_S and Cd(CH_3_COO)_2_ (4) Hydrothermal 160 °C for 24 h	H_2_O	0.25 M Na_2_S and 0.35 M Na_2_SO_3_	9679 μmol/h/g_cat._	Cd(CH_3_COO)_2_, CH_4_N_2_S, MoS_2_	Chen et al. [80]
0.4%Ti_2_C/g-C_3_N_4_	Ti_2_C flakes (NH_4_F 16 g/HCl 9 M, 24 h)	(1) Stirring ethanol (2) 550 °C, 4 h in muffle	H_2_O	10 vol% TEOA	950 μmol/h/g_cat._	Melamine	Shao et al. [81]
10%TiO_2_@C/g-C_3_N_4_	Multilayer Ti_3_C_2_ (HF 49%, 4 h)	(1) Stirring in water (2) 550 °C, 2 h in muffle	N_2_	20 vol% methanol	250 μmol/h/g_cat._	Melamine	Liu et al. [19]
Pd-Ti_3_C_2_/g-C_3_N_4_(1: 10)	Multilayer Ti_3_C_2_ (HF 40%, 24 h)	(1) Grinding (2) 500 °C, 2 h in muffle (3) Pd electrodeposition	CO_2_	0.1 M KHCO_3_	25,100 μmol/h/g_cat._	Thiourea	Xu et al. [82]
0.001 molTiO_2_/Ti_3_C_2_	Multilayer Ti_3_C_2_ (HF 49%, 24 h, 60 °C)	(1) Stirring (2) Hydrothermal 180 °C, 18 h	Methyl orange (MO)	–	40 mg/L/h	TiSO_4_	Gao et al. [83]
TiO_2_/5%Ti_3_C_2_	Ti_3_C_2_ flakes (HF 48%, 15 h and DMSO delamination, 15 h)	(1) Stirring in ice-water bath (2) Heated 95 °C, 4 h	H_2_O	25% methanol	43 μmol/h/g_cat._	TiCl_4_	Wang et al. [84]
TiO_2_/5%Ti_2_C	Ti_2_C flakes (HF 10%, 10 h and DMSO delamination)						
TiO_2_/5%Nb_2_C	Nb_2_C flakes (HF 48%, 90 h and 20% isopropyl alcohol delamination)						
CdS/2.5%Ti_3_C_2_	Ti_3_C_2_ nanoparticles (HF 49%, 20 h, 60 °C and H_2_O delamination, ultrasonication, 5 h)	(1) Stirring in water (2) Hydrothermal 180 °C, 12 h	H_2_O	Lactic acid (88 vol%)	14,342 μmol/h/g	Cd(Ac)_2_	Ran et al. [70]
						Thiourea	
TiO_2_/C/BiVO_4_ (1: 1079)	Ti_3_C_2_ flakes (LiF 1.5 g/HCl 6 M, 48 h, 50 °C)	(1) Stirring in water (2) Hydrothermal 100 °C, 6 h	RhB	–	3.1 mg/L/h	Bi(NO_3_)_3_	Shi et al. [85]
						NH_4_VO_3_	
TiO_2_/Ti_3_C_2_ (1: 1)	Multilayer Ti_3_C_2_ (HF 40%, 26 h, 60 °C)	(1) Stirring in 10 M NaOH (2) Hydrothermal 180 °C, 10 h	Methylene blue (MB)	–	8.5 mg/L/h	P25	Luo et al. [86]
BiOBr/Ti_3_C_2_ (250: 1)	Ti_3_C_2_ flakes (LiF 3 g/HCl 9 M, 24 h, 35 °C)	(1) Stirring (2) Refluxed 80 °C, 2 h	RhB	–	24 mg/L/h	Bi(NO_3_)_3_ and KBr	Liu et al. [87]
2%Ti_3_C_2_/Bi_2_WO_6_	Ti_3_C_2_ flakes (HF 40%, 72 h and DMSO delamination, ultrasonication, 1 h)	(1) Stirring (2) Hydrothermal 120 °C, 24 h	CO_2_	–	2.22 μmol/h/g_cat._	Bi(NO_3_)_3_	Cao et al. [88]
						Na_2_WO_4_	
Bi_0.9_Gd_0.1_Fe_0.8_Sn_0.2_O_3_/Ti_3_C_2_	Multilayer Ti_3_C_2_ (HF 39%, 36 h)	(1) Stirring in 0.01 M acetic acid and ethylene glycol (2) Sonicated, 2 h, 60 °C (3) stirring 1 h, 80 °C	Congo red	–	–	Bi_1−*x*_Gd_*x*_Fe_1−*y*_Sn_*y*_	Tariq et al. [89]
In_2_S_3_/TiO_2_@ Ti_3_C_2_T_*x*_ (1: 0.123)	Multilayer Ti_3_C_2_ (HF 50%, 20 h)	(1) Stirring (2) Hydrothermal 180 °C, 24 h	MO	–	18 mg/L/h	In(NO_3_)_3_	Wang et al. [90]
						CH_3_CSNH_2_	
ZnS/0.75 wt%Ti_3_C_2_	Ti_3_C_2_ flakes (HF, 24 h, 25 °C)	(1) Stirring in ethanol–glycerol (2) Hydrothermal 180 °C, 10 h	H_2_O	20 vol% lactic acid	502.6 μmol/h/g_cat._	ZnCl_2_	Tie et al. [91]
Ti_2_C/3%TiO_2_/1%Ag	Multilayer Ti_2_C (HF 48%)	(1) Stirring for volatiles evaporation (2) Annealing in H_2_ at 400 °C	Salicylic acid	–	32.4 μmol/h	Titanium isopropylate	Wojciechowski et al. [92]
TiO_2_/Ti_3_C_2_ (12 h)	Multilayer Ti_3_C_2_ (HF 49%, 12 h, 60 °C)	Hydrothermal 160 °C for different time, NaBF_4_ and HCl	MO	–	24 mg/L/h	–	Peng et al. [71]
TiO_2_/Ti_3_C_2_ (20 h)	Multilayer Ti_3_C_2_ (HF 49%, 12 h, 60 °C)	Hydrothermal 200 °C for different time, NH_4_F	MB	–	6 mg/L/h	–	Peng et al. [93]
HC-TiO_2_	Ti_3_C_2_ flakes (tetramethylammonium hydroxide 25%, 24 h)	Hydrothermal 160 °C, 9 h	H_2_O	10 vol% TEOA	33.04 μmol/h/g_cat._	–	Jia et al. [94]
4%Cu_4_/TiO_2_@Ti_3_C_2_T_*x*_-12 h	Multilayer Ti_3_C_2_ (HF 49%, 12 h, 60 °C)	(1) Hydrothermal 160 °C for different time, NaBF_4_ and HCl (2) Photodepositing copper nanodots	H_2_O	6.7 vol% methanol	764 μmol/h/g_cat._	–	Peng et al. [95]
Ti_3_C_2_/TiO_2_/CuO (100:1)	Multilayer Ti_3_C_2_ (HF 49%, 24 h, 60 °C)	(1) Dissolved in water (2) Annealing in argon, 500 °C, 30 min	MO	–	15 mg/L/h	–	Lu et al. [96]
C/TiO_2_-700 °C-150 sccm	Multilayer Ti_3_C_2_ (HF 40%, 90 h, 55 °C)	Heated in CO_2_ at different temperature and different rate, 1 h	H_2_O	10 vol% TEOA	480 μmol/h/g_cat._	–	Yuan et al. [97]
TiO_2_/Ti_3_C_2_ (TT550 °C)	Multilayer Ti_3_C_2_ (HF 50%, 48 h)	Calcination at different temperature	CO_2_	–	4.4 μmol/h/g_cat._	–	Low et al. [98]
Nb_2_O_5_/C/Nb_2_C-1 h	Multilayer Nb_2_C (HF 50%, 90 h)	Annealing in CO_2_, 850 °C for different time	H_2_O	25% methanol	7.81 μmol/h/g_cat._	–	Su et al. [99]
Microporous-MXene/TiO_2−*x*_ nanodots	Multilayer Ti_3_C_2_ (HF 50%, 90 h)	30% H_2_O_2_, 10 min	RhB.etc.	–	–	–	Cheng et al. [100]
C/TiO_2_	Multilayer Ti_2_C (HF 40%, 2.5 h)	High-energy ball milling in air, 1.5 h, 200 rpm	MB	–	2.13 mg/L/h	–	Li et al. [101]
TiO_2_/Ti_3_C_2_@AC-48 h	Multilayer Ti_3_C_2_ (HF 49%, 24 h)	Heated in H_2_O for different time at 60 °C	H_2_O	29 g/L ascorbic acid (AA)	33.4 μmol/h/g_cat._	–	Sun et al. [102]
Ti_3_C_2_/TiO_2_-500/Pt	Multilayer Ti_3_C_2_ (HF 40%, 72 h)	(1) Hydrothermal in 1 M NaOH and 30% H_2_O_2_, 140 °C, 12 h (2) Immersed in 0.1 M HCl, 24 h (3) Annealing in muffle for different time	H_2_O	20 vol% methanol	H_2_ 1596.35 μmol/h/g_cat._	–	Li et al. [103]
				0.01 M AgNO_3_	O_2_ 500 μmol/h/g_cat._		
				–	H_2_ 526 μmol/h/g_cat._ and O_2_ 315 μmol/h/g_cat._		
LDC-S-TiO_2_/C	Multilayer Ti_3_C_2_ (HF 40%, 48 h, 45 °C)	(1) Ball mixing with sulfur (2) Hydrothermal 155 °C,12 h (3) Annealing in CO_2_ at 700 °C for 2 h (4) Annealing in air at 450 °C, 2 h	H_2_O	10% methanol	333 μmol/h/g_cat._	–	Yuan et al. [104]
TiO_2_/Ti_3_C_2_	Multilayer Ti_3_C_2_ (HF 30%, 10 h, 40 °C)	Hydrothermal 160 °C for 12 h, NaBF_4_ and HCl	Carbamazepine	–	1.48 mg/L/h	–	Shahzad et al. [105]
Ti_3_C_2_/TiO_2_/15%MoS_2_	Multilayer Ti_3_C_2_ (HF 40%, 72 h)	(1) Hydrothermal 160 °C for 12 h with NaBF_4_ and HCl (2) Hydrothermal 200 °C for 24 h with Na_2_MoO_4_ and CN_2_H_4_S	H_2_O	TEOA	6425 μmol/h/g_cat._	NaBF_4_, HCl, Na_2_MoO_4_ and CN_2_H_4_S	Li et al. [106]

### In Situ Decoration of Semiconductors onto the Surface of MXenes

**Fig. 5:**
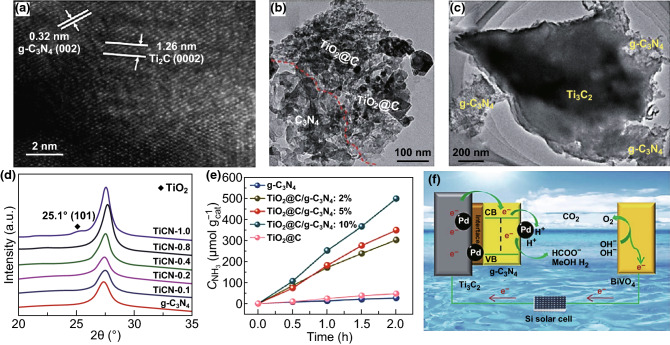
Photocatalytic composites based on MXene in combination with g-C_3_N_4_ formed by in situ decoration. **a**–**c** TEM images, **d** XRD patterns, **e** hydrogen production, and **f** mechanism for PEC reduction of CO_2_ from Shao et al. **a**, **d** Reprinted with permission from Ref. [81]. Copyright 2017 The Royal Society of Chemistry; **b**, **e** with permission from Ref. [19], Copyright 2018 The Royal Society of Chemistry; and **c**, **f** with permission from Ref. [82]. Copyright 2018 The Royal Society of Chemistry

In contrast to composites prepared by mechanical mixing of materials, in situ decoration methods consist in synthesizing a different material directly onto the MXene surface. As a result, in situ synthetized materials and MXenes are chemically bonded, which could be an important advantage in some designs. However, the range of viable synthetic conditions for in situ decoration is limited, because MXenes are easily oxidized in solution, especially at high temperatures [107]. It is therefore necessary to use mild conditions to protect MXenes from oxidation, especially when mono- and few-layered MXenes are used. So far, g-C_3_N_4_, TiO_2_, CdS, and bismuth compounds have been bonded to various MXenes using this strategy.

g-C_3_N_4_ is one 2D semiconductor material that is combined with MXenes used as a co-catalyst in the photocatalysis process (Fig. 5). MXene can be added during the calcination of a precursor, such as melamine and thiourea, but the high calcination temperature (around 550 °C) may cause the oxidation of MXene into TiO_2_. The high photoactivity of g-C_3_N_4_/MXene is attributed to the efficient charge separation; moreover, the heterojunction formed by TiO_2_/g-C_3_N_4_ also plays an important role in charge separation [108]. Shao et al. [81] synthesized Ti_2_C/g-C_3_N_4_ by melamine calcination and used it in hydrogen production (Fig. 5a, d). Though the ratio of Ti_2_C in the composite was as low as 0.4 wt%, a peak due to TiO_2_ resulting from the oxidation of Ti_2_C could be seen in the XRD pattern. Liu et al. [19] synthesized TiO_2_@C/g-C_3_N_4_ heterojunction by melamine calcination (Fig. 5b), where Ti_3_C_2_ was oxidized to TiO_2_@C during the calcination process. This composite was highly effective in the reaction of nitrogen reduction to ammonia, with the best performance reaching as high as 250.6 μmol h^−1^ g^−1^, which was better than that of TiO_2_@C and g-C_3_N_4_ (Fig. 5e). Xu et al. [82] synthesized Ti^3+^-rich Ti_3_C_2_/g-C_3_N_4_ by calcination of thiourea and employed it as an electrode for CO_2_ reduction in a photoelectrocatalytic (PEC) system (Fig. 5c, f), achieving a total CO_2_ reduction rate of 25.1 mmol h^−1^ g^−1^. The Ti^3+^ species suppressed charge recombination at the Ti_3_C_2_/g-C_3_N_4_ heterojunctions, leading to a corresponding increase in CO_2_ conversion efficiency.

**Fig. 6:**
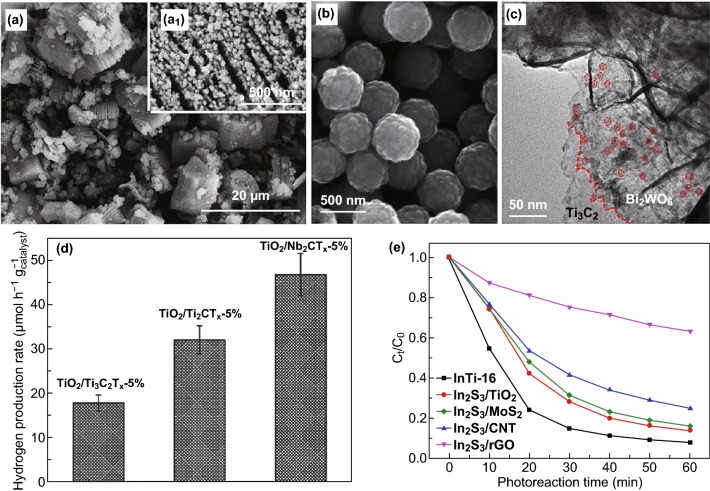
Photocatalysts based on in situ decoration of MXenes. SEM images from **a** Gao et al. Reprinted with permission from Ref. [83]. Copyright 2015 Elsevier. **b** Ran et al. Reprinted with permission from Ref. [70]. Copyright 2017 Nature Publishing Group. **c** TEM images from Cao et al. Reprinted with permission from Ref. [88]. Copyright 2018 John Wiley & Sons. **d** Hydrogen production from Wang et al. Reprinted with permission from Ref. [84]. Copyright 2016 John Wiley & Sons. **e** Degradation of methyl orange (MO) from Wang et al. Reprinted with permission from Ref. [90]. Copyright 2018 Elsevier

Apart from the above-mentioned synthesis methods, composite photocatalysts can also be synthesized by combining TiO_2_, a metal sulfide, or a bismuthide with MXene under hydrothermal conditions (Fig. 6). Gao et al. [83] synthesized TiO_2_/Ti_3_C_2_ nanocomposites by a hydrothermal method using TiSO_4_ as a precursor for methyl orange (MO) degradation (Fig. 6a), where small TiO_2_ particles could be observed on the surface of multilayered Ti_3_C_2_. Wang et al. [84] employed TiCl_4_ as the precursor in the hydrothermal synthesis of rutile TiO_2_/Ti_3_C_2_T_*x*_ for hydrogen production by water splitting (Fig. 6d). The photocatalytic activity of TiO_2_ when combined with other MXenes (Ti_2_CT_*x*_ and Nb_2_CT_*x*_ flakes) as co-catalysts was also explored; results proved that in general, MXenes could be used as effective co-catalysts for solar hydrogen production. Ran et al. [70] combined CdS and Ti_3_C_2_ particles by a one-step hydrothermal reaction (Fig. 6b). A hydrogen production rate of 14,342 μmol h^−1^ g^−1^ was achieved when using Ti_3_C_2_ as the co-catalyst; this performance is 136.6 times higher than that of the pure CdS photocatalyst. The effectivity and versatility of Ti_3_C_2_ MXene as a co-catalyst for photocatalytic hydrogen production was demonstrated by other metal sulfides (ZnS) [91] photocatalysts as well. Xie et al. [73] showed that Ti_3_C_2_ flakes enabled the local confinement of Cd^2+^ released during photo-corrosion and thus enhanced the stability of the metal sulfide. Besides CdS, In_2_S_3_/Ti_3_C_2_T_*x*_ hybrids synthesized by hydrothermal method have been used for methyl orange degradation as reported by Wang et al. [90]. Among the hybrids based on other additives (carbon nanotubes (CNT), rGO, MoS_2_, and TiO_2_), Ti_3_C_2_-based composites showed the best photocatalytic activity, which is attributed to their high electrical conductivity. Shi et al. [85] synthesized TiO_2_/C/BiVO_4_ composites by hydrothermal method for the degradation of Rhodamine B, where Ti_3_C_2_ was employed both as a support for the growth of BiVO_4_ nanoparticles and as a precursor for the generation of 2D-carbon upon oxidation. The electron transfer process was accelerated by the presence of Ti_3_C_2_-derived 2D-carbon layers, thus improving the photocatalytic performance for Rhodamine B degradation. Ultrathin 2D/2D heterojunction of MXene/Bi_2_WO_6_ prepared by the in situ growth of ultrathin Bi_2_WO_6_ nanosheets on the surface of ultrathin Ti_3_C_2_ nanosheets for photocatalytic CO_2_ reduction was reported by Cao et al. [88] (Fig. 6c). The CH_4_ and CH_3_OH yield were 4.6 times higher than those obtained with pristine Bi_2_WO_6_, which was ascribed to the enhanced CO_2_ adsorption arising from the increased specific surface area and improved pore structure of the layered heterojunction. The different composites/hybrids containing MXene or MXene-derived products prepared by hydrothermal methods and used in photocatalysis are listed in Table 1.

The synthetic process for MXenes-based composites includes doping into the photocatalysts or using MXene as a support for in situ decoration of the semiconductor photocatalyst. The chemical reactions taking place during photocatalyst formation led to increased interfacial area, thus providing greater possibilities for the transfer of photogenerated electrons. However, one disadvantage of this method is the oxidation of MXenes during photocatalyst synthesis. Although difficult to precisely characterize, conditions of formation of the photocatalysts may be too harsh and cause structural degradation of MXenes, especially in the case of single-layered MXenes, due to their lower stability toward oxidation.

### MXene-Derived Photocatalysts

**Fig. 7:**
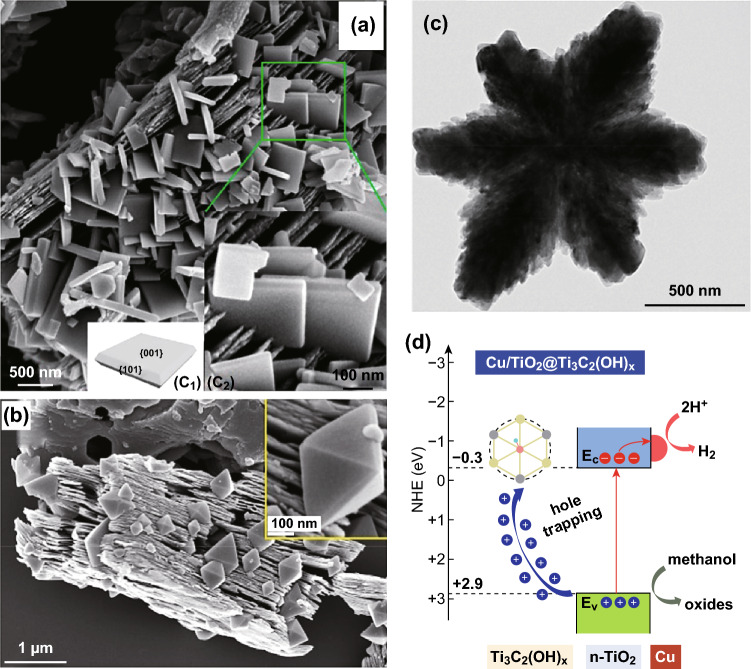
In situ oxidized MXenes by hydrothermal method for photocatalysis. SEM images taken from **a** Peng et al. Reprinted with permission from Ref. [71]. Copyright 2016 American Chemical Society. **b** Peng et al. Reprinted with permission from Ref. [93]. Copyright 2017 Elsevier. **c** TEM image from Jia et al. Reprinted with permission from Ref. [94]. Copyright 2018 American Chemical Society. **d** Charge transfer in Cu/TiO_2_@Ti_3_C_2_(OH)_*x*_ from Peng et al. Reprinted with permission from Ref. [95]. Copyright 2018 Elsevier

Different from mechanical mixing, self-assembly, and decoration methods, the in situ oxidation method using MXene (Ti_3_C_2_ is the most studied example) as a precursor for the synthesis of photocatalysts has also been explored (Fig. 7). Peng’s group tuned the facet of TiO_2_/Ti_3_C_2_ using a hydrothermal method without using an additional TiO_2_ precursor (Fig. 7a, b) [71, 93]. NaBF_4_ and NH_4_F were used as reagents to, respectively, control morphology in the synthesis of (001) TiO_2_/Ti_3_C_2_ and (111) TiO_2_/Ti_3_C_2_, which were then applied in methyl orange degradation. Both the facet type of TiO_2_ and the ratio of TiO_2_ to Ti_3_C_2_ could be controlled by changing the duration of the hydrothermal reaction. Jia et al. [94] obtained closely aggregated TiO_2_ nanorods with high carbon doping starting from Ti_3_C_2_ flakes and demonstrated a better photoactivity than commercially available P25 for hydrogen production (Fig. 7c). The carbon doping also changed the electron structure of TiO_2_ and enhanced its light absorption ability. Peng et al. [95] also used Ti_3_C_2_ as a hole trap and Cu as an electron trap to separate the charges through a dual-carrier-separation mechanism, showing the potential of MXene as an efficient functional material for photocatalysis (Fig. 7d).

**Fig. 8:**
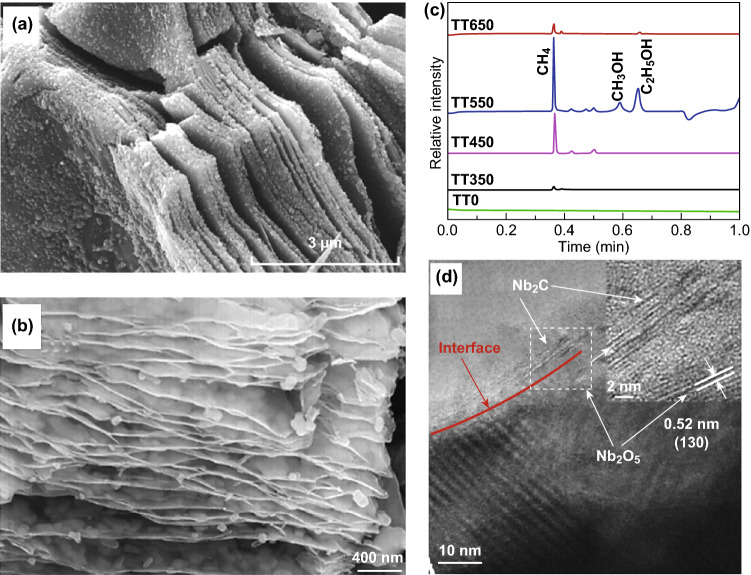
Photocatalysts containing in situ oxidized MXenes formed by calcination. SEM images from **a** Lu et al. Reprinted with permission from Ref. [96]. Copyright 2017 Hindawi. **b** Yuan et al. Reprinted with permission from Ref. [97]. Copyright 2017 John Wiley & Sons. **c** Gaseous products of CO_2_ reduction from Low et al. Reprinted with permission from Ref. [98]. Copyright 2018 Elsevier. **d** TEM image from Su et al. Reprinted with permission from Ref. [99]. Copyright 2018 John Wiley & Sons

Calcination under atmosphere containing gases such as CO_2_ and O_2_ is another method used for the controlled oxidation of MXenes (Fig. 8). Lu et al. [96] obtained Ti_3_C_2_/TiO_2_/CuO by annealing Cu(NO_3_)_2_ and Ti_3_C_2_ together under argon atmosphere (Fig. 8a). Because of its good electronic conductivity, the incorporation of Ti_3_C_2_ improved electron/hole separation and led to better methyl orange degradation. Yuan et al. [97] annealed Ti_3_C_2_ in CO_2_ to prepare 2D-layered C/TiO_2_ hybrids used in hydrogen production, in which the presence of 2D carbon layers increased electron transport channels and enhanced charge separation efficiency (Fig. 8b). In addition, the effects of oxidation temperature and CO_2_ on the grain size and crystal structure of TiO_2_ were also investigated, revealing that increasing oxidation temperature and CO_2_ gas flux led to larger grain sizes and more rutile TiO_2_ formation. Low et al. [98] calcined Ti_3_C_2_ at different temperatures, enabling the in situ growth of TiO_2_ nanoparticles on Ti_3_C_2_ nanosheets, thus forming TiO_2_/Ti_3_C_2_ composites with different loading amounts of TiO_2_ with the aim to improve performance in CO_2_ reduction reaction (Fig. 8c). Interestingly, three main products were obtained during the photocatalytic CO_2_ reduction process due to the sufficiently high intrinsic reduction potential of TiO_2_. Results of the study also pointed out that excess of Ti_3_C_2_ in the composite could have an adverse effect on photocatalytic performance. Su et al. [99] used CO_2_ to partially oxidize Nb_2_C to form Nb_2_O_5_/Nb_2_C composites for hydrogen production, where Nb_2_O_5_ and metallic Nb_2_C served, respectively, as the semiconductor photocatalyst and co-catalyst (Fig. 8d). The easily formed junction at the interface served as an electron sink to efficiently capture photogenerated electrons and suppress recombination of photogenerated electron–hole pairs, thus enhancing the efficiency of charge separation and contributing to improved photocatalytic activity [71, 93, 99, 102].

**Fig. 9:**
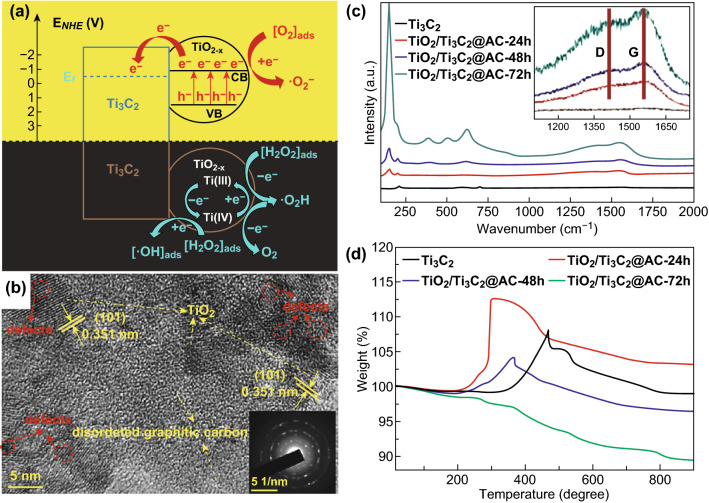
MXene-derived photocatalysts synthesized by other in situ oxidation methods. **a** Mechanisms of degradation over mp-MXene/TiO_2-*x*_ from Cheng et al. Reprinted with permission from Ref. [100]. Copyright 2018 The Royal Society of Chemistry. **b** TEM image from Li et al. Reprinted with permission from Ref. [101]. Copyright 2018 Elsevier. **c** Raman spectra and **d** TGA from Sun et al. Reprinted with permission from Ref. [102]. Copyright 2018 The Royal Society of Chemistry

Besides the hydrothermal method and calcination, other routes such as chemical oxidization and high-energy ball milling were also used to oxidize MXenes (Fig. 9). Cheng et al. [100] oxidized Ti_3_C_2_ flakes with 30% H_2_O_2_ to form microporous-MXene/TiO_2−*x*_ nanodots (Fig. 9a). This composite worked as a photo-Fenton bifunctional catalyst for Rhodamine B degradation under both dark and illumination conditions. Li et al. [101] synthesized TiO_2_@C nanosheets from Ti_2_C by high-energy ball milling and used it for methylene blue degradation (Fig. 9b). Shortly thereafter, our group used water to oxidize Ti_3_C_2_ to be applied in hydrogen production using Eosin Y as a sensitizer [102]. Similar to other oxidized MXenes, amorphous carbon and TiO_2_ were formed after oxidation (Fig. 9c, d). The various MXene-derived composites obtained by in situ oxidation to be used as photocatalysts are listed in Table 1.

The MXenes oxidation is different from other methods because of the residual presence of carbon (mostly amorphous carbon) after oxidation, and the M element is oxidized into metal oxide on the carbon layer. Thus, the composite obtained is of the form metal oxide/MXenes/C. Both MXenes and C can be used as co-catalysts in the photocatalysis process. However, in this method, the ratio of the photocatalyst to MXenes varies within a certain range since no precursor is introduced. The limitation of this method is that only a few semiconductors (depending on M element) can be used as the photocatalyst.

## Mechanism of MXenes as Co-catalysts

**Fig. 10:**
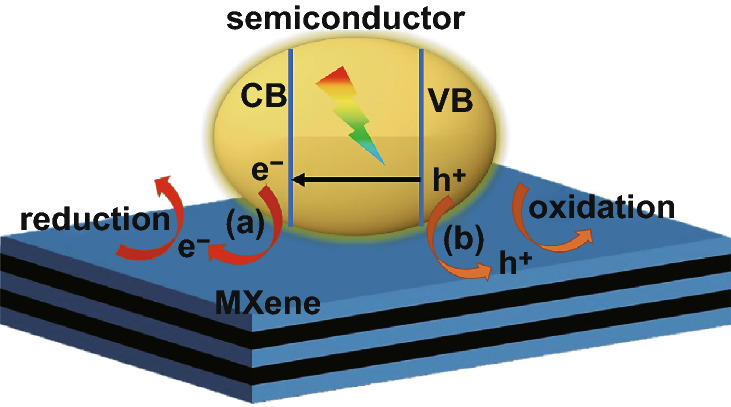
Schematic of the working mechanism of MXenes applied in photocatalysis

Since MXenes are conductors and serve as co-catalysts, the mechanism of action of a MXenes-based photocatalytic system is through accelerated charge separation and suppression of carrier recombination [69–71]. The photocatalysts absorb visible light and photogenerated electrons are excited to the CB, while holes are left in the valence band (VB). The excited charge carriers are transferred to MXenes at the interface mainly because of the higher potential of MXenes. Electrons transfer to MXenes without recombination and react on the MXene surface to generate H_2_ by reducing H^+^ [74, 78, 81, 91, 94, 102, 103], CH_4_ and CO by reducing CO_2_ [88, 98], or NH_3_ by reducing N_2_ [19], as shown in Fig. 10 process (a). In process (b), holes transfer to MXenes and react to produce OH· that can be utilized for degradation of organics [71, 93, 95]; electrons can also produce OH· for organic degradation [71, 93]. The charge transfer process from the photocatalyst to MXenes improves electron–hole pair separation and suppresses charge recombination in photocatalysts, thus enhancing the photoactivity.

Another advantage of using MXenes in photocatalysis is due to their termination groups. For example, –O termination groups show the best potential for hydrogen production because of their low |Δ*G*_H_| and the availability of active sites for the adsorption of hydrogen atoms [70, 74]. Though termination groups are important in photocatalysis, currently, it has not been possible to precisely control the relative concentrations of the different termination groups. Using presently available synthetic methods, changing the different reaction conditions can partially modify the termination groups on MXenes surface and thereby affect their performance in photocatalysis.

## Conclusion and Outlook

In summary, the application of MXenes in photocatalysis has shown rapid development since 2015. Among the MXenes family, Ti_3_C_2_ has been the most studied MXene. Mechanical mixing and self-assembly are mild and easy methods of synthesis, where the ratio of MXenes to the photocatalyst can be controlled. In addition, MXenes can also be doped into the photocatalysts by in situ decoration of a semiconductor photocatalyst. The large interfacial area afforded by the doping process improves electron transfer. However, the MXenes oxidation method has the advantage of obtaining both carbon and MXenes as co-catalysts by forming a metal oxide/MXenes/C structure. Though the above-mentioned four synthetic methods are generally used for photocatalysts, with further development in the field of MXenes, new processes may be discovered.

Besides developing improved synthetic methods, the other aspects that need to be focused on in the future are as follows:

1. Controlling the morphologies of MXenes. MXene flakes show larger surface area than multilayered MXenes, since mono- or few-layered MXenes provide a greater number of active sites for photocatalytic reactions. The flakes are also convenient for building structures, such as quantum dots, spheres, and nanorods. However, the instability of MXenes should be taken into account during heat treatment [107].
2. MXenes combine with efficient photocatalysts. MXenes can be used as co-catalysts to combine with many semiconductor photocatalysts due to their excellent electronic conductivity and the presence of numerous hydrophilic groups on the surface. Hundreds of semiconductor photocatalysts have been reported for photocatalysis so far. Attention should be paid to combining the efficient and cheap photocatalysts with MXenes to achieve better photocatalytic performance. So far, only g-C_3_N_4_, CdS, ZnS, TiO_2_, CuO, Nb_2_O_5_, BiVO_4_, Ag_3_PO_4_, α-Fe_2_O_3_, In_2_S_3_, Bi_2_WO_6_, Bi_0.90_Gd_0.10_Fe_0.80_Sn_0.20_O_3_, and BiOBr have been explored, with TiO_2_ and g-C_3_N_4_ attracting the most attention.
3. Surface modification of MXenes. Surface termination groups significantly affect the properties of MXenes, and thus, tuning the surface termination groups and modifying the MXenes surface are expected to greatly influence its potential as co-catalyst.
4. Synthesis of new MXenes. To date, only a small fraction of the different possible MXenes has been synthesized in laboratories. Some MXenes showing semiconducting properties have been reported based on theoretical calculations. Theoretical predictions help in the synthesis of semiconductor MXenes and applied in photocatalysis. Once obtained experimentally, potential MXenes can be applied as photocatalysts, thus widening the application range of MXenes. Moreover, new types of transition metal borides (MBenes) have also been predicted [34, 109] and have shown potential for photocatalysis applications. More work needs to be done in this direction.
5. Developing new synthesis methods for MXenes. HF and in situ HF wet chemical treatment are by far the most used methods in MXenes synthesis. Other HF-free methods are emerging and leading to MXenes with different properties. Yet, these have not been investigated in photocatalytic applications, and thus, the effect of the type of synthesis process used on the final performance of the MXene is currently not understood.

In short, due to tremendous effort of scientists worldwide, the great potential of MXenes in photocatalysis has been revealed. With the fast-growing development in this area, it is expected that more and more studies will focus on the applications of MXenes photocatalysis and pave the way to the commercialization of photocatalytic technologies based on these materials.
